# Environmentally friendly, but behaviorally complex? A systematic review of e-scooter riders’ psychosocial risk features

**DOI:** 10.1371/journal.pone.0268960

**Published:** 2022-05-31

**Authors:** Sergio A. Useche, Adela Gonzalez-Marin, Mireia Faus, Francisco Alonso

**Affiliations:** 1 ESIC Business & Marketing School, Valencia, Spain; 2 Department of Economic and Legal Sciences, University Center of Defense, Santiago del la Ribera, Spain; 3 INTRAS (Research Institute on Traffic and Road Safety), University of Valencia, Valencia, Spain; Shahrood University of Technology, ISLAMIC REPUBLIC OF IRAN

## Abstract

**Introduction:**

E-scooters have made a place for themselves on urban roads as an affordable, easy-to-use and environmentally friendly method of transportation. However, and partly because of their road behaviors and safety outcomes, e-scooter users have started to represent a focus of attention for transport planners and policymakers.

**Aim:**

The present systematic review aims to target and analyze the existing studies investigating the psychosocial characteristics of e-scooter riders, focusing on their behavioral and risk-related features.

**Methods:**

For this systematic review, the PRISMA methodology was used, which allows for the selection of suitable papers based on the study topic, in accordance with a set of pre-defined criteria and a search algorithm. A total of 417 indexed articles were filtered, resulting in only 32 eligible original articles directly addressing the issue. WOS, Scopus, NCBI, Google Scholar, and APA databases were used to create and test search techniques.

**Results:**

At the literature level, most of the existing studies are distributed in a few regions of the globe. At the user’s level, results show how e-scooters are most commonly used by young, highly educated, urban-dwelling males, usually for short trips. In regard to road behavior, individuals with the lowest degrees of risk perception remain more prone to engaging in risky road behaviors likely to increase their crash involvement. This might be worsened by the lack of normative e-scooter regulations (and their enforcement) in many countries, plus the marked absence of road training processes. As common limitations, it can be mentioned that 87.5% of these studies used self-report methods, while 59.4% had local coverage.

**Conclusions:**

The findings of this systematic review endorse the growing need to develop and enforce traffic laws and training processes for e-scooter users. In addition, road safety education and training programs are highlighted by existing studies as potentially pertinent alternatives to increase risk perception, and reduce risky behaviors, road conflicts and crash likelihood among e-scooter riders.

## Introduction

Urban policymakers have faced different challenges linked to transportation sustainability, efficiency, safety, and security during recent decades. For instance, road fatalities, environmental pollution and transport densification remain active threats to road users worldwide [1, 2]. Alongside a rapid transformation of transport dynamics, ‘micromobility’ stands out as a very attractive option for many people [3]. Indeed, recent studies, apart from denoting its unexpected growth, highlight its effects on travel patterns, users’ behavior and community health [4, 5].

Among all personal mobility vehicles (PMVs), and especially in urban areas, the most popular devices are–at present–electronic scooters, usually known as *e-scooters* [6, 7]. Normally, e-scooters are relatively cheap and affordable electric devices available in (foldable) small size, light weight (commonly between 17–30 lbs., or 8–13 kg), and low maintenance costs. They can, however, reach considerable speeds with low energy consumption [4, 7]. Further, and given their capability to facilitate commuting by avoiding traffic jams and interchangeably using different types of roads, the e-scooter trade has been strengthened by its “environmentally friendly” features, as it is considered a sustainable and low-polluting means of transport, of course as long as it is used correctly [8].

In addition, electric scooter sharing has increased the number of people using e-scooters [9]. Shared e-scooters can be picked up and dropped off anywhere within a commonly wide service area. In this regard, their convenience and flexibility have led to electric scooter sharing changing mobility dynamics. In fact, the evidence indicates that shared e-scooters have grown more rapidly than any other type of shared urban micro-mobility vehicle [10]. In other words, many cities (as well as many of their inhabitants) have adopted both private and shared e-scooters over the last few years. They were encouraged by potential benefits such as decreasing travel time, making trips cheaper, reducing carbon dioxide (CO_2_) emissions and avoiding massive transport means, especially during the COVID-19 pandemic [3, 11].

Notwithstanding all their benefits, the existing literature addressing PMVs remarks on some common drawbacks for e-scooter users:

In the first place, most of the current e-scooter riders do not count on specialized training for their operation. In other words, driver training and licensing procedures are not commonly required for their acquisition and usage. In addition, many of these riders are young adults, which already places them as a ‘risk’ profile according to road safety figures [4, 5].

Secondly, some studies argue that the features of PMVs might make e-scooter riders think that it is a ‘harmless toy’, making them even more likely to perform road risky behaviors [2, 6].

Thirdly, it is worth highlighting that PMVs have several limitations in terms of passive safety, which often depends almost totally on the use of appropriate protective devices and wearables. However, the state of affairs in terms of legal knowledge and enforcement is of considerable concern [5, 12]. Accordingly, it is known that head contusions and fractures are e-scooter riders’ most common injuries [13].

Fourthly, and despite (*i*) the frequent problem of accident underreporting, and (*ii*) that they cannot be directly compared to numbers of drivers and pedestrians, e-scooter-related crash figures keep rising in different countries. For instance, 1,500 crashes involving e-scooters were registered between 2017 and 2019 in the United States [14]. Meanwhile, in Spain, only during the year 2020, there were more than 100 severe crashes involving e-scooter riders [15].

Another usual constraint highlighted by studies is legislation, or the lack of it. In many regions, the novelty of e-scooters means that there is still no national legislation regulating the rules of the road for these devices in most countries. At worst, these laws remain unknown to users or unenforced by authorities [16]. In addition, in low-regulated areas, and since e-scooters cannot be fully identified with plates, sanctions for risky behavior are considerably scarce.

Indeed, some sources suggest that these feelings of anonymity and unpunishability might enhance the likelihood of several generic traffic violations, such as red-light running, riding on the pavement, speeding, zig-zagging, riding while intoxicated, getting too close to pedestrians and vehicles, and not wearing helmets [5, 12, 17].

Another huge ambiguity is where e-scooters should circulate. Preliminary, technical studies on this means of transport tend to report contrasting results regarding, among others, the most appropriate place for their circulation, i.e., sidewalks, conventional roads, bike lanes, or all of them [4, 18]. This has led some government entities to have contradictory opinions about, on the one hand, promoting environmentally-friendly transport solutions and, on the other, guaranteeing users’ road safety, especially in the absence of any type of road education or training [18]. Although still scarce, a few studies seem to coherently endorse the need of fostering laws and human behavioral-based solutions for facing this emerging challenge for road safety, especially in the case of some pioneer countries with a more developed state of affairs in this matter, including Australia, France or Singapore where e-scooter regulations do actually exist, showing some promising results [4, 19].

Some of these advances can be seen in a previous recent review study conducted by Orozco-Fontalvo, Llerena & Cantillo [20], analyzing the existing literature on e-scooters but with a special focus on e-scooter usage trends, prices, regulations and their environmental impact. Interestingly, this systematic review highlights (apart from their absence) several deficiencies in the application of laws and regulations regarding electric scooters. Another interesting finding of this study is related to the user profile: their findings indicate that e-scooter riders were predominantly young people (especially men), who previously used sustainable transport modes, especially bicycles, even though other relevant issues such as riders’ psychosocial characteristics, road behavior and risk-related issues remain pending to be addressed.

### Study aim

Bearing in mind the aforementioned considerations, the aim of this systematic review was to target and analyze the existing studies investigating the psychosocial characteristics of e-scooter users, focusing on their behavioral and risk-related features. For this purpose, a set of data source search criteria, which will be detailed below, was defined to properly retrieve/analyze the most relevant studies made and provide relevant insights for strengthening further research and policymaking in this field.

## Methods and materials

### Study approach

Overall, systematic reviews can be understood as a method of mapping the current literature by successively following a transparent and systematic approach to establish a research topic, discover studies, assess their quality, and synthesize findings, either qualitatively or statistically [21]. Also, and far from necessarily seeking the ability to generalize findings (that is something that often inexperienced researchers mistakenly look for in a systematic review), this method focuses on describing the state of affairs on a certain topic or research question to the best of the possibilities.

For this research, the core recommendations provided by Arksey and O’Malley’s methodology for systematic reviews of the literature were followed to explain and improve each stage of the framework [22]. The five stages are as follows:

1. Identifying the Research Question,
2. Finding Relevant Studies,
3. Selecting the Studies,
4. Charting the Data and Collating,
5. Summarizing and Reporting the Results.

#### Step 1: Identifying the research question

As previously mentioned, the present systematic review aims to target and analyze the existing studies investigating the psychosocial characteristics of e-scooter users and their behavioral and risk-related features. As specialized literature commonly endorses the idea that risky road (human) behavior constitutes the main predictor of crashes [23, 24], it is important to explore the characteristics of e-scooter users as an emerging group of users in cities according to their demographic profiles. In this sense, we seek to explore the main themes of the studies on e-scooter users as well as the possible discrepancies (or concordances) of the results.

As this does not constitute a meta-analytic experience, no statistical comparisons were made. Also, it is worth remarking that, as in many other "emerging" research topics, it is common to find that some regions are underrepresented in terms of scientific production in this field. A summary and topic analysis of all the selected research articles were included in the final report.

#### Step 2: Finding relevant studies

The current review was conducted in accordance with the PRISMA standards for systematic review notification. PRISMA is the acronym for “Preferred Reporting Items for Systematic Reviews and Meta-Analyses”, and it is an evidence-based method that establishes a set of items for reporting in systematic reviews [25].

PRISMA starts the process with the records identified in the searches carried out in each of the different databases. It continues with the total number of records after duplicates are eliminated and ends with the individual studies in the qualitative and quantitative synthesis [26]. This methodology, which allows following a structured (but still flexible) set of steps, has been widely used in other studies and systematic reviews on various topics, including human behavior and traffic crashes in the case of different groups of road users [27–30].

The databases that were used to conduct a preliminary literature search were the Web of Science, American Psychological Association (APA), Scopus, National Center for Biotechnology Information (NCBI) and Google Scholar. These databases were selected because of the large number of articles they store and their relationship to behavioral-based studies, especially from the fields of psychology/behavioral sciences and applied road safety [31, 32].

Other lists of systematic and comprehensive reviews of other primary research publications, which were theoretically eligible but not collected by our search algorithms, were also examined. This was carried out in order to target potentially suitable studies not indexed within the aforementioned data sources.

The search covered publications from the beginning of the database, and included the literature published in the accessed indexes/databases up to January 2022. Among the terms, we looked for were: “e-scooter users”, “e-scooter riders”, “e-scooter”, “attitudes”, “behavior” (and “behaviour”), “safety”, “risk perception”, “road risk perception” “user profile” “and “road users”. We came up with these terms, after reviewing the titles and keywords of the articles we found during our preliminary search,.

Based on what is advised in specialized literature [33–35], our research criteria comprised the application of three essential and widely used Boolean operators: "AND", to jointly retrieve results over terms whose main value for the systematic review lies in the fact of being used in the same study (e.g., e-scooters, riders); "OR", especially to emphasize on potentially similar terms that should vary in terms of writing and naming, but not in meaning (e.g., behavior/behaviour); and "NOT", to exclude frequently observed, clearly irrelevant results (e.g., price, regulations).

#### Step 3: Selecting the studies

During this stage, articles that did not refer to our research goal were eliminated, excluding articles on e-scooters with a technical or technological profile and only including user-focused research, usually of a more psychological or sociological nature. Conference/summaries, protocols, letters, editorials, case reports, and case series were not among the considered publications. We also limited our eligibility criteria to publications published in English and Spanish that were either publicly available or could (at worst) be requested through the library system, making them able to be analyzed.

A subset of titles and summaries was initially examined by each author independently, and the results were tallied. This constitutes a common process in the case of systematic reviews addressing road behavioral-based research. It covers the case of different groups of users and/or compares them, as it has been done during recent years with the case of reviews on cyclist behaviors [36, 37].

Another relevant clarification worth to be made is that, given our focus on behavioral and risk-related factors and not on e-scooter crash consequences, issues such as road trauma and riders’ fatalities were not included in our search criteria, albeit these topics could be among the various topics addressed by the studies to analyze.

#### Step 4: Charting the data

The descriptive-analytic method of Arksey & O’Malley was used to critically review the papers that matched the inclusion criteria [22]. The following information was retrieved and recorded for each eligible article: author(s), year of publication, country of study, study design, group of users investigated, sample size, key findings, and highlighted results, as well as their core limitations and shortcomings. Other previous systematic reviews in the field of road users’ behavior report similar information from the selected articles [38, 39].

#### Step 5: Collating, summarizing, and reporting the results

Since this helps to increase the chances of adequately categorizing, unifying and diagramming the information for comparative purposes (favoring the data understanding among readers), the descriptive data were evaluated using a thematic-based organization technique after the graphed data were summarized in tables. For this purpose, the main sections and issues used for empirical research were summarized in successive columns (see Table 1).

**Table 1:** Structured sescription of study setting, key outcomes and limitations of eligible studies.

Author(s) and year	Country	Study aim(s) and setting	Users/Sample	Method	Results (key outcomes)	Key limitations
***E-scooter riders’ basic features and trip-related dynamics***						
Nikiforiadis et al., 2021 [40]	Greece	The study was designed based on 578 questionnaires to identify the characteristics of e-scooters users.	E-scooter riders (n = 271) and non-e-scooter users (n = 307)	Cross-sectional and Observational	Shared e-scooters mostly replaced sustainable transport modes. Bicycle or motorcycle users were not at all attracted by e-scooters. Male and urban areas riders have a greater interest in using e-scooters.	(1) Self-report (2) Local coverage
Fitt & Curl, 2019 [41]	New Zealand	The research presents a survey on the attitudes and use of e-scooters.	E-Scooters and non-e-scooter users (n = 591)	Cross-sectional and Observational	71% of participants had used an e-scooter, 29% had not. Younger people, men and those in full-time employment are the most likely to use e-scooters. Safety concerns and expenses top the list of practical reasons for not using an e-scooter.	(1) Self-report (3) Low reliability (4) Not representative
Christoforou et al., 2021 [42]	France	The study presents the design and results of an extensive face-to-face road survey among e-scooter users.	E-scooter riders (n = 459)	Cross-sectional and Observational	E-scooters users are mostly men, aged 18–29, with a high educational level. Their main motivation is travel time, playfulness and money savings. They shifted mainly from walking and public transportation.	(1) Self-report (5) Small sample
Ratan et al., 2021 [43]	United States	The research examines how perceptions of e-scooter mobile apps influence intent to use e-scooters.	E-scooter riders and non-e-scooter users (n = 398)	Cross-sectional and Observational	Mobile app perceived ease of use is associated with e-scooter use intent. This effect is mediated by e-scooter perceived usefulness, even when controlling for e-scooter perceived ease of use and other influential elements e-scooter use.	(1) Self-report (2) Local coverage (4) Not representative (5) Small sample
Buehler et al., 2021 [44]	United States	This study reports results from attitudes and preferences of e-scooter riders and non-users using two cross-sectional surveys deployed before and after the launch of a fleet of shared e-scooters.	E-scooter riders (n = 428) and non-e-scooter users (n = 462)	Cross-sectional and Observational	Perceptions about convenience, cost, safety, parking, rider behavior, and usefulness of the e-scooter systems were more positive among non-riders after the system launch. Participants want more bike lanes or separate spaces for electric scooters.	(1) Self-report (2) Local coverage
Sanders et al., 2020 [45]	United States	This paper characterize trends in the barriers and benefits related to e-scooter.	E-Scooter riders and non-e-scooter users (n = 1,256)	Cross-sectional and Observational	E-scooters are seen as a convenient way to travel. African American and non-white Hispanic respondents were more likely to try e-scooters and to be unhappy with current transportation options. E-scooters are associated with concerns about traffic safety.	(1) Self-report (2) Local coverage (6) Not exhaustive
Almannaa et al., 2021 [46]	Saudi Arabia	The study explores the feasibility of launching an e-scooter sharing system as a new micro-mobility mode.	E-scooter riders and non-e-scooter users (n = 439)	Cross-sectional and Observational	Results showed that most of the Saudi community is unfamiliar with e-scooter systems. Most of those who have ridden e-scooters before have tried them outside Saudi Arabia.	(1) Self-report (4) Not representative (5) Small sample
Kopplin et al., 2021 [47]	Germany	To reveal factors affecting e-scooter usage from a consumer’s perspective, a study using an adapted Unified Theory of Acceptance and Use of Technology.	E-Scooter riders and non-e-scooter users (n = 749)	Cross-sectional and Observational	E-scooters are mostly viewed as fun objects, and perceived safety indeed impedes their usage. Environmental concerns and individual convenience evince to represent the main drivers for using e-scooters.	(1) Self-report (4) Not representative
Huang & Lin, 2018 [48]	Taiwan	To understand the potential needs of scooter riders and provide product/service design suggestions for increasing user willingness to accommodate e-scooters.	E-scooter riders (n = 190)	Cross-sectional and Observational	Scooter design and usage can evoke positive emotions. Pragmatic quality and individual differences of gender and age have been found to influence scooter usage.	(1) Self-report (4) Not representative (5) Small sample
Fitt & Curl, 2020 [49]	New Zealand	This paper draws on an online survey completed by residents cities in which shared electric scooters.	E-Scooters and non-E-Scooters (n = 491)	Cross-sectional and Observational	Changes in the materials, competencies, and meanings associated with urban mobility as a response to the e-scooter trial.	(1) Self-report
Hyvönen, Repo & Lammi, 2016 [50]	Finland	The research analyzes and characterizes future uses of light electric vehicles.	E-Scooter riders and non-e-scooter riders (n = 1,030)	Cross-sectional and Observational	Consumers show interest in electric vehicules and the paper addresses the match between different kinds of consumers and these vehicles, building opportunities for large scale use.	(1) Self-report
Zhang et al., 2021 [51]	United States	This study develops an e-scooter route choice model to reveal riders’ preferences for different types of transportation infrastructures.	E-scooter riders (n = 76,652 e-scooter trips-GPS)	Cross-sectional and Observational	E-scooter riders are willing to travel long distances to ride on bikeways, multi-use paths, tertiary roads, and one-way roads. E-scooter users also prefer shorter and simpler routes.	(1) Self-report (7) Data limitations
Bieliński & Ważna, 2020 [52]	Poland	This article compares the features of users of e-bike and e-scooter sharing systems and travel behavior.	Cyclists and e-scooter riders (n = 632)	Cross-sectional and Observational	E-bikes are utilized for first- and last-mile transportation, and to commute straight to various points of interest. In turn, e-scooters are primarily used for leisure riding.	(1) Self-report (2) Local coverage
Flores & Jansson, 2021 [53]	Denmark	This study determines how users and non-users perceive the shared e-vehicles, and how CI influences the adoption of shared micro vehicles.	E-scooter riders (n = 1,501)	Cross-sectional and Observational	Users see shared micro vehicles as somewhat green, whereas non-users do not. When comparing users’ perceptions of shared e-bike use, CI and green perceptions are associated to shared e-bike use, whereas only CI is linked to shared e-scooter use.	(1) Self-report (2) Local coverage (4) Not representative
Mitra & Hess, 2020 [54]	Canada	The paper investigates residents’ self-reported intentions to consider shared e-scooters.	E-scooter riders (n = 1,640)	Cross-sectional and Observational	21% were open to using e-scooters for some of their present excursions, while the majority would use shared e-scooters to replace their existing walking (60%) and transport (55%) trips.	(1) Self-report
***E-scooter riders’ behavior***						
Taleqani & Hough, 2020 [55]	United States	This paper investigates the frequency and perceived severity of 20 risky behaviors.	Cyclists and e-scooter riders	Cross-sectional and Observational	Participants perceive there is a low risk associated with reckless behaviors.	(1) Self-report (2) Local coverage (4) Small sample
Haworth, Schramm & Twisk, 2021 [56]	Australia	The research examines illegal and risky behaviors, and interactions with pedestrians. Shared and private e-vehicles were compared.	E-scooter riders (n = 686)	Cross-sectional and Observational	Illegal riding was more prevalent among shared than private e-scooters. Non-use of helmets was more common among riders of shared e-scooters and shared bicycles than private bicycles.	(1) Self-report (2) Local coverage (3) Low reliability (8) Biased data
Brunner et al., 2020 [57]	Germany	The research analysed e-scooter stability (impact of hand signals and rear blind spot checks).	E-scooter riders	Cross-sectional and Experimental	Even novice e-scooter riders can successfully learn to maintain stability while performing these tasks.	(2) Local coverage
Rodon & Ragot-Court, 2019 [58]	China	The study compared the riding behaviors of different types of e-vehicles.	E-scooter riders (n = 400)	Cross-sectional and Observational	A continuous increase in the incidence of risky behaviors as the weight and power of vehicles increase. E-bikes appear to be different from traditional bikes and E-scooters are not significantly different from other motorized vehicles.	(1) Self-report (2) Local coverage
Bai et al., 2015 [59]	China	The study compares risky behaviors in crossing signalized intersections.	Cyclists and e-scooter riders	Cross-sectional and Observational	Compared to e-bike and bicycle riders, e-scooter riders are more likely to take risky behaviors (ride in motorized lanes and ride against traffic).	(2) Local coverage (4) Not representative
Tuncer et al., 2020 [60]	Sweden	The study analysed how e-scooter riders and pedestrians deal with the unexpected appearance of e-scooters via displays of attention, adjustments of speed and the relative rights.	E-scooter riders	Cross-sectional and Observational	Details how the surprise appearance of e-scooters to pedestrians is managed, and the e-scooter riders’ use of gaze, speed, and category-relevant spaces.	(2) Local coverage
Brown et al., 2020 [61]	United States	This research investigates the parking practices as well as the frequency and types of parking violations of e-scooters, bikes, and motor vehicles.	Cyclists, e-scooter riders, and drivers (n = 3666)	Cross-sectional and Observational	Motor vehicles impede access far more than bikes and e-scooters. Motor vehicles often impeded other travelers’ access when dropping off or picking up people or food while double parking, parking in “No Parking” areas, or blocking driveways.	(2) Local coverage (4) Not representative (9) Not generalizable
Arellano & Fang, 2019 [62]	United States	The study included observations from streets, sidewalks, and a mixed-use path (pedestrians and cyclists allowed, but no cars).	E-scooter riders (n = 330)	Cross-sectional and Observational	Males ride faster, and vary less by the facility. E-scooter riders travel slightly slower than cyclists. Helmets are uncommon among e-scooter riders. E-scooter riders are less distracted by cell phones and headphone use.	(2) Local coverage (4) Not representative (5) Small sample (9) Not generalizable
Siebert et al., 2021 [63]	Germany	To evaluate the impact of ergonomics on the safe usage of shared e-scooters and to analyze riders’ knowledge and self-reported behavior.	E-scooter riders (n observation = 2972 and n survey = 156)	Cross-sectional and Observational	Braking system design has a noticeable effect, with more riders preparing the left-hand brake than the right hand or foot brake (depending on the e-scooter model).	(5) Small sample (7) Data limitations
Siebert et al., 2021 [64]	Germany	12.5 hours of observation for helmet wear, dual-use, type of infrastructure used, and travel direction correctness.	E-scooter riders (n = 777)	Cross-sectional and Observational	One in ten e-scooter riders rode in the opposite direction of traffic. 5.1% of shared e-scooters were found to be in use twice. None of the riders wore a helmet while riding e-scooters.	(2) Local coverage (5) Small sample
***Risk perception and other psychosocial risk-related issues***						
James et al., 2019 [65]	United States	The study analysed the perceived safety around riders of e-scooters and experiences of sidewalks blocked by e-scooters.	Pedestrians and e-scooter riders (n = 181)	Cross-sectional and Observational	Respondents generally felt less safe while walking around dockless e-scooters than they were around the different types of bicycles.	(1) Self-report (2) Local coverage (5) Small sample
Che, Lum & Wong, 2020 [66]	Singapore	Virtual reality shows the perceived degree of safety, anger, and celerity of movement in six scenarios.	Pedestrians (n = 30) and e-scooter riders (n = 30)	Cross-sectional and Experimental	Pedestrians rated ES speeds of 10 km/h and 15 km/h as safer than 20 km/h in overtaking maneuvers, while 15 km/h was rated as safest in face-to-face interactions; the pattern of risk perception is positively correlated with anger levels.	(5) Small sample (9) Not generalizable
Maiti et al., 2019 [67]	United States	The research investigates crowd-sensed encounter data between e-scooters and pedestrian participants on two university campuses.	Pedestrians and e-scooter riders	Cross-sectional and Observational	The analysis uncovered encounter statistics, mobility trends and hotspots which were then used to identify potentially unsafe spatio-temporal zones for pedestrians.	(1) Self-report (2) Local coverage (4) Not representative
Kuo et al., 2019 [68]	Singapore	The study presents tbe pedestrians’ attitudes towards the use of PMDs on a shared path, the intention to use, ease of use, usefulness, perceived risk and environment.	E-Scooter riders and non-e-scooter users (n = 303)	Cross-sectional and Observational	Prior PMD riding experience does not affect the subject’s degree of acceptance of PMDs on a shared path. A high correlation was found between the environment and intention to use, as well as the perceived risk.	(1) Self-report (2) Local coverage
Löcken et al., 2020 [69]	Germany	The research analyzes the perception of users about the degree of safety of hand signals.	E-Scooter riders (n = 10 and n = 24)	Cross-sectional and Observational	The results showed a significant number of inexperienced e-scooter users. The perceived safety and skill in the behaviors performed increased with the user experience.	(1) Local coverage (2) Not representative (5) Small sample
Currans et al., 2022 [70]	United States	The study analyzes the safety behavior of e-scooter users based on the road infrastructure characteristics.	E-Scooter riders	Cross-sectional and Observational	Behaviors perceived as safe are correlated with lower accident rates. Users who prefer to ride on sidewalks were more likely to have been in a collision with other users or vehicles.	(1) Self-report (2) Local coverage
Derrick, 2020 [71]	Singapore	This study used an online survey to identify perceived safety issues posed by e-scooters.	E-Scooter riders and non-e-scooter users (n = 310)	Cross-sectional and Observational	The majority of participants perceives e-scooters as dangerous. This negative perception was minimized if the user had never used e-scooters.	(1) Self-report (2) Local coverage (5) Small sample

## Results

**Fig 1:**
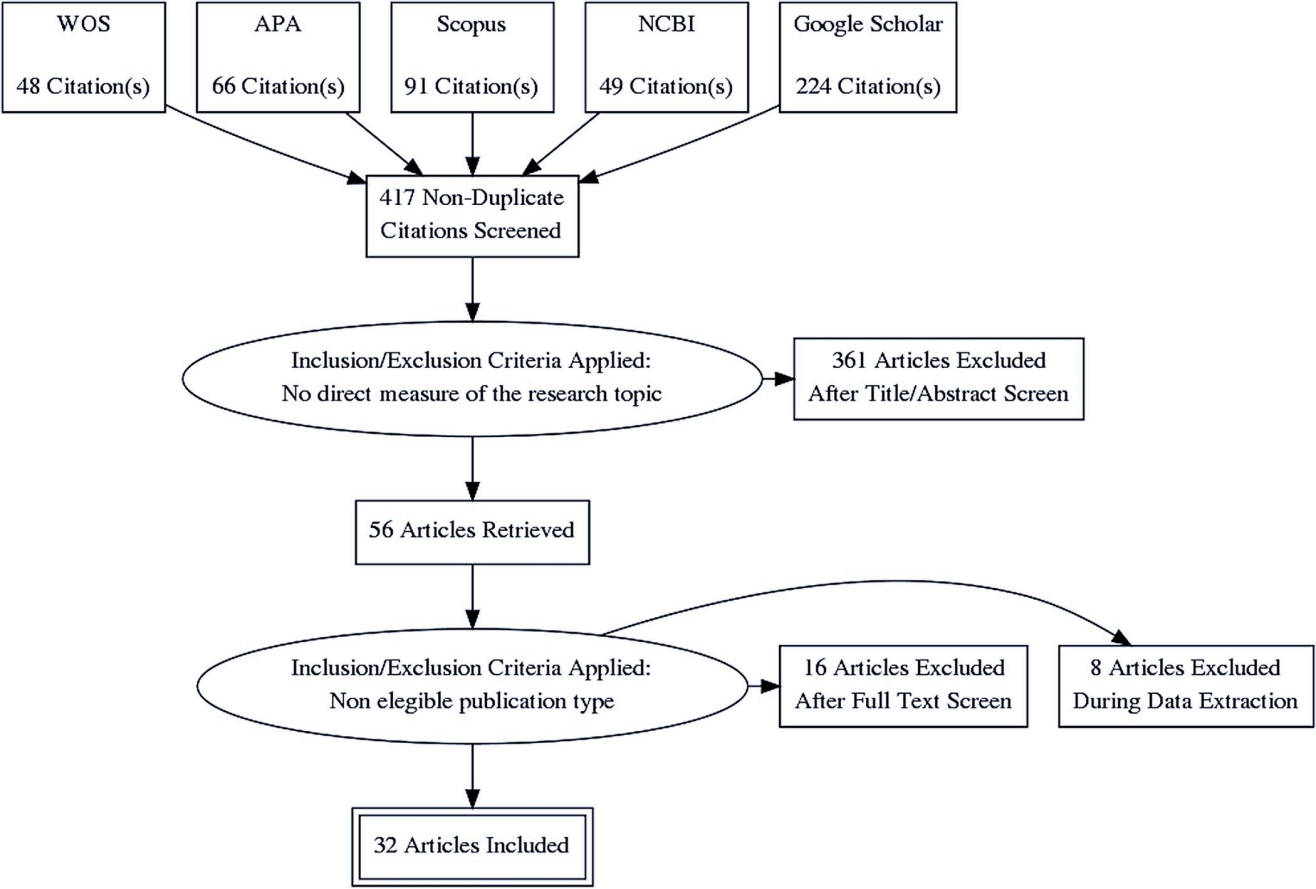
PRISMA diagram appending non-duplicate search results according to the different data sources. Abbreviations: WOS (Web of Science); APA (American Psychological Association); NCBI (National Center for Biotechnology Information).

The database search initially returned 478 possible papers able to be analyzed. However, once the doubled (paper duplicates) or non-accessible items were dismissed from the research process, the search criteria yielded a total of 417 possible outcomes after applying this filter. Also, and to avoid discarding potentially useful information sources as a consequence of indexing issues, we used a manual selection process to find papers fitting the review’s goal, ending up with 32 papers that were qualified under the aforementioned criteria. The description of the data sources and selection procedures used is graphically presented in the flowchart (Fig 1).

### Search results

#### Characteristics of eligible research articles

**Fig 2:**
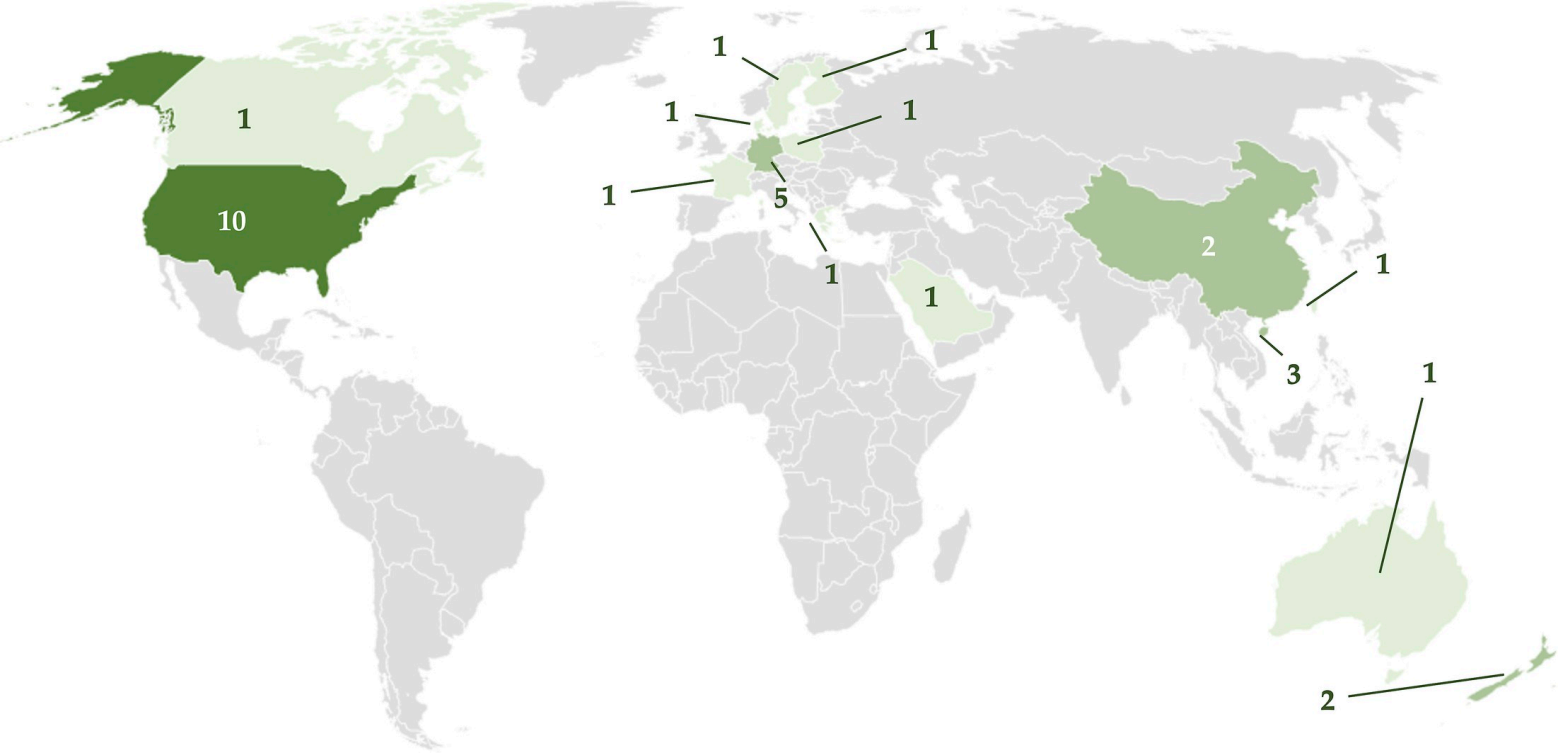
Geographical distribution (country of origin) of the selected studies (number of studies per country).

Since (and as mentioned before) this research problem has been raised within the last decade, we did not set a time frame for our search. Following this logic guideline, we found 32 papers that met the minimum inclusion criteria and were published in English between 2015 and 2022, indicating that the study topic is current. Furthermore, the research was carried out in a variety of countries. Thus, at the same time it highlights the scarcity of original studies on this subject in almost all the countries around the globe. There were 15 countries represented (ordered from highest to lowest number of empirical studies available) in the literature search results: United States (n = 10), Germany (n = 5), Singapore (n = 3), New Zealand (n = 2), China (n = 2), Australia (n = 1), France (n = 1), Greece (n = 1), Saudi Arabia (n = 1), Sweden (n = 1), Taiwan (n = 1), Poland (n = 1), Denmark (n = 1), Canada (n = 1) and Finland (n = 1), as shown in Fig 2.

It stands out that observational/cross-sectional studies most frequently address user-related features and trip dynamics (n = 15). In the same way, users’ features-related studies commonly perform comparisons among road users (n = 10). Germany is the country where more empirical studies solely focused on e-scooter riders’ behavior have been performed so far (n = 4).

Regarding methodological features of the selected eligible studies, it is worth pointing out that all of them were empirical-based and (more specifically) followed a cross-sectional design, and the vast majority are also observational, usually–although not exclusively–through online surveys or face-to-face interviews (n = 30 of the studies fulfilled this characteristic). There are only two articles where an experimental methodology is used. In both, virtual reality devices are used to expose subjects to various situations controlled by the researchers in order to observe their behaviors and decisions under such circumstances.

In all these selected research articles throughout the review process, the focus of the core study was directly related to e-scooter riders’ psychosocial issues. However, in some of them, apart from being able to gather data about psychosocial features of e-scooter users themselves, we also have the assessments of other road users on critical matters such as perceived safety, observed risk behaviors or interaction between users. Thus, in more detail, the investigations are as follows: evaluate only e-scooter riders (n = 15), compare them with non-e-scooter users (n = 10), pedestrians (n = 3), cyclists (n = 3) and with drivers and cyclists (n = 1).

#### Analysis of studies

In order to meet our core study aim, the studies were analyzed in consideration of both the PRISMA protocol guidelines [25], as well as other scoping reviews previously published, that dealt with similar and/or compatible topics or applied studies [20, 22]. These sources suggested reporting very general issues of the studies (e.g., authors, year) and their study setting, key results, and even limitations.

Although all of the studies selected approached psychosocial issues among e-scooter riders regarding their behaviors, risks and safety, the aims of these sources are very heterogeneous, so it was important to group them for the case of this systematic review. For this purpose, studies were categorized according to their core aims, albeit some secondary aims could have overlapped. This strategy allowed us to divide them into three blocks of studies, as presented in Table 1: (*i*) Studies aimed at profiling e-scooter users, i.e., e-scooter riders’ basic features and transport dynamics (n = 15); (*ii*) Those targeting to examine e-scooter users’ riding behavior, attitudes and interaction with other road users (n = 10); and (*iii*) Researches assessing risk perceptions in relation to e-scooter riders (n = 7).

## Discussion

The core aim of this systematic review was to target and analyze the existing studies investigating the psychosocial characteristics of e-scooter riders, focusing on their behavioral and risk-related features. Overall, all empirical papers addressing the profile of e-scooter users tend to highlight similar demographic, *i*.*e*., gender and age group-based characteristics, regardless of the country in which their research was conducted. A wide-ranging synthesis of the approached studies’ outcomes shows how e-scooter users are more likely to be males, young, highly educated and/or working full time, and living in urban areas. Additionally, they are often regardless of income levels, given that e-scooters are considered affordable even for the case of Low and Middle-Income Countries, or LMICs [41, 42].

### Usage and trip-related patterns in e-scooter riding

Regarding travel patterns of e-scooter users in literature, the sources selected allow to affirm that, overall, e-scooter trips tend to have a relatively short length. This fact is usually attributable to their energy storage capacity, which still remains somewhat limited. Further, the still low potential of e-scooter for longer trips seems to be–in addition to geographical and infrastructural shortcomings–the main motive why it is not as widely used by those living in peripheral or rural areas [51, 72].

Accordingly, it should be noted that the environmental value of e-scooters can be considered “relative”, as it carries both benefits and constraints. For instance, while using them to replace motorized transportations means makes them “environmentally friendly”, their usage may limit the active mobility of (e.g.) people potentially performing short urban trips by walking or cycling [20]. Further, frequent bicycle and motorcycle users are those reporting themselves as more reluctant to change their commuting habits, including shifting to e-scooters for their urban trips [40].

Another factor constantly observed in the analyzed papers is, in brief, the growing association between Information and Communication Technologies (ICTs) and the use of e-scooters. ITCs refer to the set of technologies that allow access, production, processing, treatment, storage, transmission and communication of information. Although this was not a main study variable across the studies analyzed, it is worth highlighting that, overall, the usage of new technologies tends to be (in literature) positively associated with both the intention to use e-scooters and a greater intensity of use [48, 52, 53]. Once considering this issue, it seems to fit the exposed user profile coherently: as it becomes cheaper and it easily interacts with ICT using patterns, young people who at the same time tend to be the most familiar with connected technologies and perceive the lesser risks on them and remain the most prone to adopt ‘connected’ devices for everyday mobility [43].

Accordingly, previous studies in this regard have shown that key technological developments applied to mobility tend to elicit more positive attitudes. Thus, they explain a greater intention of being adopted among individuals having higher degrees of previous interaction with other technologies. At the same time, they remain more open to new technology-related experiences [73, 74], but also more prone to assume new (and sometimes greater) levels of risk [75]. This is precisely another key outcome to be subsequently discussed in this study.

### Risk-related perceptions, assumptions and outcomes

Firstly, it is worth addressing the “troublesome” issue of subjective risk perceptions. Despite having been widely endorsed by previous studies as one of the best predictors of the intention to use both e-scooters and other PMDs, nowadays, it represents a challenge in terms of intervention. This is due to the widespread absence of policies, programs and law requirements for road safety education and training for e-scooter riders [20, 68]. Despite this significant constraint, empirical literature already provides some highlights that endorse the need for further work on developing proper interventions and policies to increase road risk perception among e-scooter users.

For instance, it has been demonstrated how an overall greater degree of subjective risk perceptions coherently contributes to explaining the fact that females shift to e-scooters with less frequency than their male counterparts, as, on average, they perceive them with higher levels of insecurity and more vulnerable to suffer different types of victimization on-road or *transit* environments [40]. This is (consistently) their main stated reason for avoiding certain trip modalities [44], including many of the different possible risk-related scenarios usually present at urban locations [76].

Also, there is a fact that, although is consistently linked in the literature to risky road behaviors and crashes, remains relatively understudied: what could be the factors most closely explaining great risk assumptions among e-scooter riders? [46, 55, 65]. In this regard, some studies highlight that, besides age and gender issues, trip-related purposes might play a relevant role in predicting risky road behaviors, making it necessary to focus on enhancing risk perceptions in any way possible [30, 55, 65]. This means that the problem can also be understood as "circular": since it does not require a licensing course, users could be encouraged to acquire an e-scooter. Consequently, they become exempt from the need to go through a road training process, making it simpler but riskier at the same time.

Moreover, another factor that seems to make things worse is that the purpose of e-scooters has been largely *leisurized*. Therefore, PMDs tend to be often perceived as "fun" devices, while their main strengths could be, instead, their (e.g.) practicality, comfort and environmental contributions [47]. In behavioral terms, it is feasible to hypothesize that this potentially problematic association between PMDs and leisure might enhance a certain *relaxation* among users, who may systematically decrease their protective habits. At the same time, their risks assumptions tend to grow, e.g., using helmets on a less regular basis, riding in the wrong way, or using sidewalks at high speeds, even for relatively short periods of their trips [12, 62].

### Could e-scooter riders’ behaviors be “the worst”?

To date, there is no accurate answer provided with a very high intercontextual validity to this question. However, some studies suggest that e-scooter users could be performing more (and more frequently) risky behaviors than cyclists and motorcyclists [59]. Some studies suggest that this could be enhanced by the disparity of opinions, laws, and directions regarding the proper regulation of the circulation of e-scooters in urban locations [5, 77].

Regarding other users’ perceptions, pedestrians have been shown to be those self-reporting the greatest feeling of unsafety when interacting with e-scooters, whose riders tend to be considered as ‘riskier’ than cyclists [66, 67]. This may be due to several reasons. For one, the novelty of e-scooters makes them unfamiliar to many people, who are much more accustomed to sharing their space with bicycles [49]. This is compounded by the high speeds (commonly up to 25km/h) these vehicles can reach, and the nature of the injuries commonly suffered in their crashes [78, 79]. Specifically, serious knee, thorax and/or head injuries are the more likely to occur in e-scooter-pedestrian crashes, the last being usually the most affected, according to hospital records [15].

As a response to this big concern, some *pioneer* efforts have been made to analyze the consequences of these crashes. For instance, research conducted in Germany [64, 80], Denmark [81], and New Zealand [82], among other countries, determined that the introduction of e-scooters in urban traffic has had a significant impact on healthcare centers. Furthermore, in Spain, an increase of 31% in injuries and 20% in deaths due to this cause has been detected in the last year [15]. In this regard, the results synthesized in this study suggest that, at the risky road behavior level, the absence of helmets can be considered as an underestimated factor. Although not preventing traffic crashes, the absence of helmets weighed most heavily in the deaths recorded in 2020, highlighting its importance as a passive safety measure [83, 84].

On the other hand, in many cities, there are lanes delimited exclusively for cyclists, aimed at reducing the chances of them invading pavement and other pedestrian locations [44]. In this regard, identifying conflict zones and delimiting safe spaces for e-scooters to minimize the risks of collisions or falls remain a crucial issue for preventing road conflicts, near-misses, and crashes involving PMD riders [67].

Altogether, studies analyzing the socio-demographic and behavioral elements of e-scooter users present similar findings, even though their number and deepness can be considered relatively scarce. This is a key limitation currently existing in the literature, consequently affecting the present review. Further, e-scooter riders’ features, dynamics and figures remain unaddressed in many geographical locations, making it difficult to provide globally reliable inferences on this topic, even though some insights are provided by the existing literature.

### Common limitations of existing studies and further research

Finally, it is worth acknowledging some limitations commonly present in the existing literature on the topic. As this is a systematic review, we will focus on the studies analyzed. In the first place, and as reported in Table 1 (see right column), the majority of research on e-scooter users’ behavior and risk-related factors uses surveys as the method par excellence for data collection, accounting for 87.5% of the research papers. While this type of design is very useful for gathering large samples and extensive datasets [85], self-reported information implies many potential biases. Among the most frequent, social desirability, stereotypes, unrealistic attributions (e.g., over- or under-estimations) or differences between respondents and non-respondents attributable to nonresponse bias stand out [86, 87]. Therefore, these study outcomes should be carefully and contextually analyzed. Also, future research could be benefited from employing other methods to complement the evidence obtained from questionnaire-based research.

Another limitation that occurs in 59.4% of the selected studies is the small territory in which they have been carried out. In many cases, the studies present a local coverage that may restrict the degree of generalizability of the results [88]. This phenomenon is exacerbated if the sample is small (34.3% of the articles selected) and/or if the questionnaires are only administered in specific areas of a locality that are not representative of the population as a whole (this represents 40.6% of the articles in the review). In these studies, biases arise from the characteristics of the population stratum analyzed (e.g., a university campus will have mostly young participants, or a particular neighborhood will have subjects of a similar socio-economic level).

To a lesser extent, the studies report limitations such as low reliability (6.25%), low exhaustiveness (3.12%) or data limitations derived from the quality of the images recorded (6.25%). In any case, all the limitations indicated do not detract from the value of the results. On the contrary, they provide greater validity and rigor to the research process developed [89]. Moreover, the accumulation of evidence provides findings and indications that should be considered in future studies covering this research field.

## Conclusions

The findings of this study show, at the literature level, that most of the existing studies are distributed in a few regions of the world (North America, Central Europe, Eastern Asia and Oceania). At the user’s level, results indicate that e-scooters are most commonly used by young, highly educated, urban-dwelling males, usually for short trips.

Further, the accumulated evidence can state that the basic particularities of e-scooter users, as well as their attitudes, behaviors, and perceptions, are similar despite the differential cultural characteristics of the countries analyzed. Notwithstanding, there is no cross-culturally validated evidence regarding the mechanisms linking e-scooter riders’ psychosocial features, behaviors, and crashes. Consequently, and at the practical level, this study shows how the existing empirical scientific production on this matter: (*i*) remains considerably scarce, and (*ii*) is reduced to a few countries (and the production is minimum in LMICs), even though e-scooter riding has grown in a global level during the last decade.

Therefore, further research addressing psychosocial and behavioral risk-related issues among e-scooter riders and other MVP users might contribute to formulating, developing and enforcing more effective regulations and actions aimed at reducing their psychosocial road risks and, therefore, their crash likelihood.

### Ethics

The Research Ethics Committee at the Research Institute on Traffic and Road safety at the University of Valencia assessed and approved the study protocol, certifying its accordance to the current ethical guidlines applicable to systematic reviews (IRB approval number RE0001160921). As this is a systematic review and there are no human participants, informed consent is not required.

## Supporting information

S1 Checklist PRISMA checklist for systematic reviews. (DOCX)

## References

[1] Brenner N, Schmid C. The ‘urban age’ in question. Int J Urban Reg Res, 2014, 38(3): 731–755. 10.1111/1468-2427.12115.

[2] Sutton JC. Gridlock: Congested cities, contested policies, unsustainable mobility. 2015. Routledge.

[3] Li A, Zhao P, Haitao H, Mansourian A, Axhausen KW. How did micro-mobility change in response to COVID-19 pandemic? A case study based on spatial-temporal-semantic analytics. Comput Environ Urban Syst, 2021, 90: 101703. doi: 10.1016/j.compenvurbsys.2021.101703 34629583 PMC8492604

[4] Glavić D, Trpković A, Milenković M, Jevremović S. The E-Scooter Potential to Change Urban Mobility—Belgrade Case Study. Sustainability, 2021, 13(11): 5948. 10.3390/su13115948.

[5] Fonseca-Cabrera AS, Llopis-Castelló D, Pérez-Zuriaga AM, Alonso-Troyano C, García A. Micromobility Users’ Behaviour and Perceived Risk during Meeting Manoeuvres. Int J Environ Res Public Health, 2021, 18(23): 12465. doi: 10.3390/ijerph182312465 34886198 PMC8656849

[6] Hollingsworth J, Copeland B, Johnson JX. Are e-scooters polluters? The environmental impacts of shared dockless electric scooters. Environ Res Lett, 2019, 14(8): 084031. 10.1088/1748-9326/ab2da8.

[7] Hosseinzadeh A, Algomaiah M, Kluger R, Li Z. E-scooters and sustainability: Investigating the relationship between the density of E-scooter trips and characteristics of sustainable urban development. Sustain Cities Soc, 2021, 6: 102624. 10.1016/j.scs.2020.102624.

[8] Gössling S. Integrating e-scooters in urban transportation: Problems, policies, and the prospect of system change. Transp Res D Transp Environ, 2020, 79: 102230. 10.1016/j.trd.2020.102230.

[9] Jiao J., & Bai S. Understanding the shared e-scooter travels in Austin, TX. ISPRS Int J Geo-Inf, 2020, 9(2): 135. 10.3390/ijgi9020135. PMC11139225 38818355

[10] Eccarius T., & Lu C. C. Adoption intentions for micro-mobility–Insights from electric scooter sharing in Taiwan. Transp Res D Transp Environ, 2020, 84: 102327. 10.1016/j.trd.2020.102327.

[11] Guo Y., & Zhang Y. Understanding factors influencing shared e-scooter usage and its impact on auto mode substitution. Transp Res D Transp Environ, 2021, 99: 102991. 10.1016/j.trd.2021.102991.

[12] Haworth NL, Schramm A. Illegal and risky riding of electric scooters in Brisbane. Med J Australia, 2019, 211(9): 412–413. doi: 10.5694/mja2.50275 31291003

[13] Ishmael CR, Hsiue PP, Zoller SD, Wang P, Hori KR, Gatto JD, et al. An early look at operative orthopedic injuries associated with electric scooter accidents: bringing high-energy trauma to a wider audience. J Bone Joint Surg Am, 2020, 102(5): e18. doi: 10.2106/JBJS.19.00390 31895168

[14] Felton R. E-Scooter Ride-Share Industry Leaves Injuries and Angered Cities in its Path. Consumer Reports. 2019. Available online: https://www.consumerreports.org/product-safety/e-scooter-ride-share-industry-leaves-injuries-and-angered-cities-in-its-path/ (accessed on 30 Jan 2022).

[15] Mapfre Fundación. Pruebas de choque (crash-test) de patinetes eléctricos y riesgos asociados a su proceso de recarga: recomendaciones para un uso seguro. 2021. Available online: https://documentacion.fundacionmapfre.org/documentacion/publico/es/catalogo_imagenes/grupo.do?path=1109839 (accessed on 26 Jan 2022).

[16] Yang H, Ma Q, Wang Z, Cai Q, Xie K, Yang D. Safety of micro-mobility: analysis of E-Scooter crashes by mining news reports. Accid Anal Prev, 2020, 143: 105608. doi: 10.1016/j.aap.2020.105608 32480017

[17] Sparks AM, Fessler DM, Zinsser M. Exploring the Roles of Conformity, Hazard, and Convenience in Risk Mitigation Decisions: An Observational Study of Helmet Use Among Bicyclists and E-scooter Riders in Los Angeles During Two Natural Experiments. 2019. 10.31234/osf.io/gspbm.

[18] RACE. Así está la normativa de patinetes eléctricos en España. 2021. Available online: https://www.race.es/patinete-electrico-legislacion (accessed on 27 Jan 2022).

[19] Yan X, Yang W, Zhang X, Xu Y, Bejleri I, Zhao X. Do e-scooters fill mobility gaps and promote equity before and during COVID-19? A spatiotemporal analysis using open big data, 2021. 10.48550/arXiv.2103.09060.

[20] Orozco-Fontalvo M, Llerena L, Cantillo V. Dockless electric scooters: A review of a growing micromobility mode. Int. J. Sustain. Transp., 2022, 1–17. 10.1080/15568318.2022.2044097.

[21] Armstrong R, Hall BJ, Doyle J, Waters E. Scoping the scope’of a Cochrane review. J Public Health, 2011, 33(1): 147–150. doi: 10.1093/pubmed/fdr015 21345890

[22] Arksey H, O’Malley L. Scoping studies: towards a methodological framework. Int J Soc Res Methodol, 2005, 8(1): 19–32. 10.1080/1364557032000119616.

[23] Puchades VM, Pietrantoni L, Fraboni F, De Angelis M, Prati G. Unsafe cycling behaviors and near crashes among Italian cyclists. Int J Inj Control Saf Promot, 2018, 25(1): 70–77. 10.1080/17457300.2017.1341931. 28675090

[24] Stephens AN, Brown J, de Rome L, Baldock MRJ, Fernandes R, Fitzharris M. The relationship between Motorcycle Rider Behaviour Questionnaire scores and crashes for riders in Australia. Accid Anal Prev, 2017, 102: 202–212. doi: 10.1016/j.aap.2017.03.007 28324820

[25] Moher D, Altman DG, Liberati A, Tetzlaff J. PRISMA statement. Epidemiology, 2011, 22(1): 128. doi: 10.1097/EDE.0b013e3181fe7825 21150360

[26] Urrútia G, Bonfill X. PRISMA declaration: A proposal to improve the publication of systematic reviews and meta-analyses. Medicina clínica, 2010, 135(11): 507–511. doi: 10.1016/j.medcli.2010.01.015 20206945

[27] Garbarino S, Guglielmi O, Sanna A, Mancardi GL, Magnavita N. Risk of occupational accidents in workers with obstructive sleep apnea: systematic review and meta-analysis. Sleep, 2016, 39(6): 1211–1218. doi: 10.5665/sleep.5834 26951401 PMC4863208

[28] Heidari M, Aryankhesal A, Khorasani-Zavareh D. Laypeople roles at road traffic crash scenes: a systematic review. Int J Inj Control Saf Promot, 2019, 26(1): 82–91. doi: 10.1080/17457300.2018.1481869 29939119

[29] Akbari M, Lankarani KB, Heydari ST, Motevalian SA, Tabrizi R, Sullman MJ. Is driver education contributing towards road safety? a systematic review of systematic reviews. J Inj Violence Res, 2021, 13(1):69. doi: 10.5249/jivr.v13i1.1592 33459279 PMC8142340

[30] Oviedo-Trespalacios O, Truelove V, Watson B, Hinton JA. The impact of road advertising signs on driver behaviour and implications for road safety: A critical systematic review. Transp Res A Policy Pract, 2019, 122: 85–98. 10.1016/j.tra.2019.01.012.

[31] Azami-Aghdash S, Aghaei MH, Sadeghi-Bazarghani H. Epidemiology of road traffic injuries among elderly people; a systematic review and meta-analysis. Bull Emerg Trauma, 2018, 6(4): 279. doi: 10.29252/beat-060403 30402515 PMC6215074

[32] Malakoutikhah M, Rabiei H, Hassanipour S, Jahangiri M. The prevalence of unsafe behaviors in Iranian workers: a systematic review and meta-analysis. Iran J Public Health, 2021, 50(2): 257. doi: 10.18502/ijph.v50i2.5338 33747989 PMC7956081

[33] Bramer W. M., de Jonge G. B., Rethlefsen M. L., Mast F., & Kleijnen J. A systematic approach to searching: an efficient and complete method to develop literature searches. J Med Libr Assoc, 2018, 106(4): 531. doi: 10.5195/jmla.2018.283 30271302 PMC6148622

[34] Scells, H., Zuccon, G., & Koopman, B. Automatic Boolean query refinement for systematic review literature search. In WWW’19: The world wide web conference, 2019, (pp. 1646–1656). 10.1145/3308558.3313544.

[35] Gusenbauer M., & Haddaway N. R. Which academic search systems are suitable for systematic reviews or meta‐analyses? Evaluating retrieval qualities of Google Scholar, PubMed, and 26 other resources. Res. Synth. Methods, 2020, 11(2): 181–217. doi: 10.1002/jrsm.1378 31614060 PMC7079055

[36] Esmaeilikia M, Radun I, Grzebieta R, Olivier J. Bicycle helmets and risky behaviour: A systematic review. Transp Res Part F Traffic Psychol Behav, 2019, 60: 299–310. 10.1016/j.trf.2018.10.026.

[37] Olivier J, Creighton P. Bicycle injuries and helmet use: a systematic review and meta-analysis. Int J Epidemiol, 2017, 46(1): 278–292. doi: 10.1093/ije/dyw153 27450862

[38] Spindler DJ, Allen MS, Vella SA, Swann C. The psychology of elite cycling: a systematic review. J Sports Sci, 2018, 36(17): 1943–1954. doi: 10.1080/02640414.2018.1426978 29346033

[39] Schönbach DM, Altenburg TM, Marques A, Chinapaw MJ, Demetriou Y. Strategies and effects of school-based interventions to promote active school transportation by bicycle among children and adolescents: a systematic review. Int J Behav Nutr Phys Act, 2020, 17(1): 1–17. doi: 10.1186/s12966-020-01035-1 33183331 PMC7661215

[40] Nikiforiadis A, Paschalidis E, Stamatiadis N, Raptopoulou A, Kostareli A, Basbas S. Analysis of attitudes and engagement of shared e-scooter users. Transport Res D-Tr E, 94, 2021: 102790. 10.1016/j.trd.2021.102790.

[41] Fitt H, Curl A. Perceptions and experiences of Lime scooters: Summary survey results. 2019. Available online: http://hdl.handle.net/10092/16336 (accessed on 28 Jan 2022).

[42] Christoforou Z, Gioldasis C, de Bortoli A, Seidowsky R. Who is using e-scooters and how? Evidence from Paris. Transport Res D-Tr E, 2021, 92: 102708. 10.1016/j.trd.2021.102708.

[43] Ratan R, Earle K, Rosenthal S, Chen VHH, Gambino A, Goggin G., et al. The (digital) medium of mobility is the message: Examining the influence of e-scooter mobile app perceptions on e-scooter use intent. Comp Hum Behav, 2021, 3: 100076. 10.1016/j.chbr.2021.100076.

[44] Buehler R, Broaddus A, Sweeney T, Zhang W, White E, Mollenhauer M. Changes in Travel Behavior, Attitudes, and Preferences among E-Scooter Riders and Nonriders: First Look at Results from Pre and Post E-Scooter System Launch Surveys at Virginia Tech. Transp Res Record, 2021, 2675(9): 335–345. 10.1177/03611981211002213.

[45] Sanders RL, Branion-Calles M, Nelson TA. To scoot or not to scoot: Findings from a recent survey about the benefits and barriers of using E-scooters for riders and non-riders. Transport Res A Policy Pract, 2020, 139: 217–227. 10.1016/j.tra.2020.07.009.

[46] Almannaa MH, Alsahhaf FA, Ashqar HI, Elhenawy M, Masoud M, Rakotonirainy A. Perception analysis of E-scooter riders and non-riders in Riyadh, Saudi Arabia: Survey outputs. Sustainability, 2021, 13(2): 863. 10.3390/su13020863.

[47] Kopplin CS, Brand BM, Reichenberger Y. Consumer acceptance of shared e-scooters for urban and short-distance mobility. Transport Res D-Tr E, 2021, 91: 102680. 10.1016/j.trd.2020.102680.

[48] Huang FH, Lin SR. A Survey of User Experience of Two Wheeler Users in Long-Term Interactions. In Congress of the International Ergonomics Association (pp. 1465–1472). 2018. Springer, Cham.

[49] Fitt H, Curl A. The early days of shared micro mobility: A social practices approach. J Transport Geogr, 2020, 86: 102779. 10.1016/j.jtrangeo.2020.102779.

[50] Hyvönen K, Repo P, Lammi M. Light electric vehicles: substitution and future uses. Transport Res Proc, 2016, 19: 258–268. 10.1016/j.trpro.2016.12.085.

[51] Zhang W, Buehler R, Broaddus A, Sweeney T. What type of infrastructures do e-scooter riders prefer? A route choice model. Transport Res D-Tr E, 2021, 94: 102761. 10.1016/j.trd.2021.102761.

[52] Bieliński T, Ważna A. Electric scooter sharing and bike sharing user behaviour and characteristics. Sustainability, 2020, 12(22): 9640. 10.3390/su12229640.

[53] Flores PJ, Jansson J. The role of consumer innovativeness and green perceptions on green innovation use: The case of shared e‐bikes and e‐scooters. J Consumer Behav, 2021, 20(6): 1466–1479. 10.1002/cb.1957.

[54] Mitra R, Hess PM. Who are the potential users of shared e-scooters? An examination of socio-demographic, attitudinal and environmental factors. Travel Behav Soc, 2021, 23: 100–107. 10.1016/j.tbs.2020.12.004.

[55] Taleqani AR, Hough J. Risk Perception of Bicycle/Scooter Riders Risky Behaviors. 2020. Available online: https://www.ugpti.org/resources/reports/downloads/surtcom20-04.pdf (accessed on 26 Jan 2022).

[56] Haworth N, Schramm A, Twisk D. Comparing the risky behaviors of shared and private e-scooter and bicycle riders in downtown Brisbane, Australia. Accident Anal Prev, 2021, 15, 105981. doi: 10.1016/j.aap.2021.105981 33549973

[57] Brunner P, Löcken A, Denk F, Kates R, Huber W. Analysis of experimental data on dynamics and behavior of e-scooter riders and applications to the impact of automated driving functions on urban road safety. In 2020 IEEE Intelligent Vehicles Symposium (IV) (pp. 219–225). IEEE. 10.1109/IV47402.2020.9304835.

[58] Rodon C, Ragot-Court I. Assessment of risky behaviors among E-bike users: A comparative study in Shanghai. Transp Res Interdiscip Perspectiv, 2019, 2: 100042. 10.1016/j.trip.2019.100042.

[59] Bai L, Liu P, Guo Y, Yu H. Comparative analysis of risky behaviors of electric bicycles at signalized intersections. Traffic Inj Prev, 2015, 16(4): 424–428. doi: 10.1080/15389588.2014.952724 25133656

[60] Tuncer S, Laurier E, Brown B, Licoppe C. Notes on the practices and appearances of e-scooter users in public space. J Transp Geogr, 2020, 85: 102702. 10.1016/j.jtrangeo.2020.102702.

[61] Brown A, Klein NJ, Thigpen C, Williams N. Impeding access: The frequency and characteristics of improper scooter, bike, and car parking. Transp Res Interdiscip Perspectiv, 2020, 4: 100099. 10.1016/j.trip.2020.100099.

[62] Arellano JF, Fang K. Sunday drivers, or too fast and too furious? Transport Findings, 2019. 10.32866/001c.11210.

[63] Siebert FW, Ringhand M, Englert F, Hoffknecht M, Edwards T, Rötting M. Braking bad–Ergonomic design and implications for the safe use of shared E-scooters. Saf Sci, 2021, 140: 105294. 10.1016/j.ssci.2021.105294.

[64] Siebert FW, Hoffknecht M, Englert F, Edwards T, Useche SA, Rötting M. Safety Related Behaviors and Law Adherence of Shared E-Scooter Riders in Germany. HCI in Mobility, Transport, and Automotive Systems (HCII 2021). Lect Notes Comput Sci, 2021, 30: 12791. 10.1007/978-3-030-78358-7_31.

[65] James O, Swiderski JI, Hicks J, Teoman D, Buehler R. Pedestrians and e-scooters: An initial look at e-scooter parking and perceptions by riders and non-riders. Sustainability, 2019, 11(20): 5591. 10.3390/su11205591.

[66] Che M, Lum KM, Wong Y. D. Users’ attitudes on electric scooter riding speed on shared footpath: A virtual reality study. Int J Sustain Transp, 2020, 1–10. 10.1080/15568318.2020.1718252.

[67] Maiti A, Vinayaga-Sureshkanth N, Jadliwala M, Wijewickrama R, Griffin GP. Impact of E-Scooters on Pedestrian Safety: A Field Study Using Pedestrian Crowd-Sensing. arXiv preprint arXiv:1908.05846. 2019. Available online: https://www.tdcommons.org/dpubs_series/2280 (accessed on 29 Jan 2022).

[68] Kuo JY, Sayeed A, Tangirala NT, Han VCY, Dauwels J, Mayer MP. Pedestrians’ acceptance of personal mobility devices on the shared path: A structural equation modelling approach. In 2019 IEEE Intelligent Transportation Systems Conference (ITSC) (pp. 2349–2354). 2019. IEEE. 10.1109/ITSC.2019.8917167.

[69] Löcken, A., Brunner, P., & Kates, R. Impact of Hand Signals on Safety: Two Controlled Studies With Novice E-Scooter Riders. In 12th International Conference on Automotive User Interfaces and Interactive Vehicular Applications (pp. 132–140), 2020. 10.1145/3409120.3410641.

[70] Currans KM, Iroz-Elardo N, Ewing R, Choi D, Siracuse B, Lyons T, et al. Scooting to a New Era in Active Transportation: Examining the Use and Safety of E-scooters. Research Report #NITC-RR-1281. Portland, OR: National Institute for Transportation and Communities (NITC), 2022. 10.15760/trec.272.

[71] Derrick WTC. Perceptions and attitudes towards micro e-scooters in Singapore. 2020. Available online: https://open-education-repository.ucl.ac.uk/681/1/Wee%2C%20Derrick.pdf.

[72] Llamazares J., Useche S. A., Montoro L., & Alonso F. Commuting accidents of Spanish professional drivers: when occupational risk exceeds the workplace. Int J Occup Saf Ergon, 2021, 27(3): 754–762. doi: 10.1080/10803548.2019.1619993 31132927

[73] Useche SA, Peñarada-Ortega M., Gonzalez-Marin A, Llamazares F. Assessing the effect of drivers’ gender on their intention to use fully automated vehicles. Appl Sci, 2021. Appl Sci, 2022, 12, 103. 10.3390/app12010103.

[74] Alonso F., Faus M., Esteban C., & Useche S. A. Is there a predisposition towards the use of new technologies within the traffic field of emerging countries? The case of the Dominican Republic. Electronics, 2021, 10(10): 1208. 10.3390/electronics10101208.

[75] Useche SA, Hezaveh AM, Llamazares FJ, Cherry C. Not gendered… but different from each other? A structural equation model for explaining risky road behaviors of female and male pedestrians. Accid Anal Prev, 2021, 150: 105942. doi: 10.1016/j.aap.2020.105942 33338915

[76] Soto J, Orozco-Fontalvo M, Useche SA. Public Transportation and Fear of Crime at BRT Systems: Approaching to the case of Barranquilla (Colombia) through Integrated Choice and Latent Variable models. Transp Res Part A Policy Pract, 2021, 155(10): 142–160. 10.1016/j.tra.2021.11.001.

[77] Prada CG, García-Andrade XC. La fragmentada regulación de los vehículos de movilidad personal: un problema común en Perú y España. Revista de Derecho, 2019, 20(1): 161–201. Available online: https://revistas.udep.edu.pe/derecho/article/view/1836 (accessed on 28 Jan 2022).

[78] Sikka N, Vila C, Stratton M, Ghassemi M, Pourmand A. Sharing the sidewalk: A case of E-scooter related pedestrian injury. Am J Emerg Med 2019, 37(9): 1807–e5. doi: 10.1016/j.ajem.2019.06.017 31201118

[79] Ma Q, Yang H, Ma Y, Yang D, Hu X, Xie K. Examining municipal guidelines for users of shared E-Scooters in the United States. Transport Res D-Tr E, 2021, 92: 102710. 10.1016/j.trd.2021.102710.

[80] Störmann P, Klug A, Nau C, Verboket RD, Leiblein M, Müller D, et al. Characteristics and injury patterns in electric-scooter related accidents—a prospective two-center report from Germany. J Clin Med, 2020, 9(5): 1569. doi: 10.3390/jcm9051569 32455862 PMC7290505

[81] Nielsen KI, Nielsen FE, Rasmussen SW. Injuries following accidents with electric scooters. Danish Med J, 2021, 68(2): A09200697–A09200697. Available online: https://ugeskriftet.dk/files/scientific_article_files/2021-01/a09200697_web.pdf (accessed on 30 Jan 2022). 33543698

[82] Bekhit MNZ, Le Fevre J, Bergin CJ. Regional healthcare costs and burden of injury associated with electric scooters. Injury, 2020, 51(2): 271–277. doi: 10.1016/j.injury.2019.10.026 31668353

[83] O’Hern S, Stephens AN, Young KL, Koppel S. Personality traits as predictors of cyclist behaviour. Accident Anal Prev, 2020, 145: 105704. doi: 10.1016/j.aap.2020.105704 32771694

[84] Curl A, Fitt H. Same, but different? Cycling and e‐scootering in a rapidly changing urban transport landscape. New Zeal Geogr, 2020, 76(3): 194–206. doi: 10.1111/nzg.12271

[85] Scheaffer R. L., Mendenhall W. III, Ott R. L., & Gerow K. G. Elementary survey sampling. Cengage Learning, 2011.

[86] Mahmood K. Do people overestimate their information literacy skills? A systematic review of empirical evidence on the Dunning-Kruger effect. Commun. Inf. Lit., 2016, 10(2): 3. 10.15760/comminfolit.2016.10.2.24.

[87] Johnson T. P., & Wislar J. S. Response rates and nonresponse errors in surveys. Jama, 2012, 307(17): 1805–1806. doi: 10.1001/jama.2012.3532 22550194

[88] Ahmad N., Boutron I., Dechartres A., Durieux P., & Ravaud P. Applicability and generalisability of the results of systematic reviews to public health practice and policy: a systematic review. Trials, 2010, 11(1): 1–9. doi: 10.1186/1745-6215-11-20 20187938 PMC2838881

[89] Walker T. J., Tullar J. M., Diamond P. M., Kohl H. W., & Amick B. C. Validity and reliability of the 8-item work limitations questionnaire. J. Occup. Rehabil. 2017, 27(4): 576–583. doi: 10.1007/s10926-016-9687-5 28025750 PMC5484749

